# Effect of interrupted time during intensity modulated radiation therapy on survival outcomes in patients with nasopharyngeal cancer

**DOI:** 10.18632/oncotarget.13713

**Published:** 2016-11-30

**Authors:** Guo-Zeng Xu, Ling Li, Xiao-Dong Zhu

**Affiliations:** ^1^ Department of Radiation Oncology, Cancer Hospital of Guangxi Medical University, Nanning, People's Republic of China

**Keywords:** nasopharyngeal cancer, interrupted time, intensity modulated radiotherapy, survival outcomes, prognosis

## Abstract

**Objective:**

To evaluate the influence of interrupted time (IT) during intensity-modulated radiotherapy (IMRT) on survival outcomes in patients with nasopharyngeal cancer (NPC).

**Materials and methods:**

515 NPC patients receiving IMRT between January 2007 and December 2011 were retrospectively reviewed. The association of IT with loco-regional failure-free survival (LRFS), progression free survival (PFS) and overall survival (OS) was assessed by univariate and multivariate analysis. The Kaplan–Meier methodology was used for survival analyses and the differences among groups were assessed by the log-rank test. The Cox model was used for evaluating the prognostic factors for LRFS, PFS, and OS.

**Results:**

The 3-year PFS and OS analysis revealed significant difference between patients with a IT >4 days and those with a IT ≤4 days (72.1% *vs* 81.9%, *p* <0.05; 80.8% *vs* 87.9%, *p* <0.05). Age (≥44 years *vs* <44 years), T classification, N classification, and IT (>4 days *vs* ≤4 days) were independent prognostic factors for PFS and OS, respectively (*p* <0.05). Only N classification acted as the prognostic factor for LRFS (*p* <0.05).

**Conclusion:**

The IT of more than 4 days during IMRT may affect survival outcomes of NPC patients. We should minimize the interruption of radiotherapy caused by any reason as much as possible.

## INTRODUCTION

Nasopharyngeal cancer (NPC) is epidemic in Southern China and Southeast Asia [1, 2]. With the advancements of radiotherapy technology, intensity modulated radiotherapy (IMRT) with/without concurrent chemotherapy has become the main modality of treatment for NPC patients [3, 4]. Interruptions during radiotherapy are difficult to be evitable because the presence of public holidays, severe treatment-related toxicity, and machinery faults. Many previous studies had reported that the prolonged time of radiotherapy had adverse effects in NPC patients receiving conventional radiotherapy [5–9]. Whether the duration of radiotherapy interruptions can affect survival outcomes has not been extensively studied in NPC patients receiving IMRT [10]. So we conducted this retrospective study to evaluate the relationship between the interrupted time (IT) and survival outcomes in NPC patients receiving IMRT.

## RESULTS

### Basic data

The characteristics of patients were shown in Table 1. 385 males and 130 females were included (M/F = 2.96/l). The most patients presented with stage III and IV (51.1%). According to statistics, there were no significant differences in age, gender, WHO type, concurrent chemotherapy, T and N classifications between patients with a IT >4 days and those with a IT ≤4 days (*p* ≥ 0.05). But patients between two groups received different types of chemotherapy (*p* <0.05).

**Table 1:** Baseline disease characteristics and demographics of patients at primary diagnosis

Characteristic	All	IT≤4days	IT>4days	P
	No (%)	No (%)	No (%)	
Total no. of patients	515(100%)	388(100%)	127(100%)	
Age, years				P=0.40
<44	247 (48.0%)	206(53.1%)	62(48.8%)	
≥44	268(52.0%)	182(46.9%)	65(51.2%)	
Sex				P=0.99
Male	385 (74.8%)	290(74.7%)	95 (74.8%)	
Female	130(25.2%)	98(25.3%)	32(25.2%)	
Histology, WHO type				P=0.05
I	4 (0.8%)	4(1.0%)	0(0%)	
II	88(17.1%)	74 (19.1%)	14 (11.0%)	
III	423(82.1%)	310 (79.9%)	113(89.0%)	
T stage				P=0.22
T1	60(11.7%)	50 (12.9%)	10(7.9%)	
T2	152(29.5%)	119 (30.7%)	33(26.0%)	
T3	169(32.8%)	121 (31.2%)	48 (37.8%)	
T4	134(26.0%)	98(25.3%)	36(28.3%)	
N stage				P=0.05
N0	58(11.3%)	52 (13.4%)	6 (4.7%)	
N1	183(35.5%)	137 (35.3%)	46 (36.2%)	
N2	249(48.3%)	182 (46.9%)	67 (52.8%)	
N3	25(4.9%)	17(4.4%)	8(6.3%)	
Chemotherapy type				
None	58(11.3%)	53 (13.7%)	5 (3.9%)	P=0.01
CCRT only	109(21.2%)	77 (19.8%)	32 (25.2%)	
CCRT with NACT or ACT	331(64.3%)	243 (62.6%)	88 (69.3%)	
NACT or ACT alone	17(3.3%)	15(3.9%)	2(2.6%)	

Abbreviations: IT= interrupted time, CCRT= concurrent chemoradiotherapy, NACT= neoadjuvant chemotherapy (ACT), ACT= adjuvant chemotherapy.

### Interrupted time

**Figure 1:**
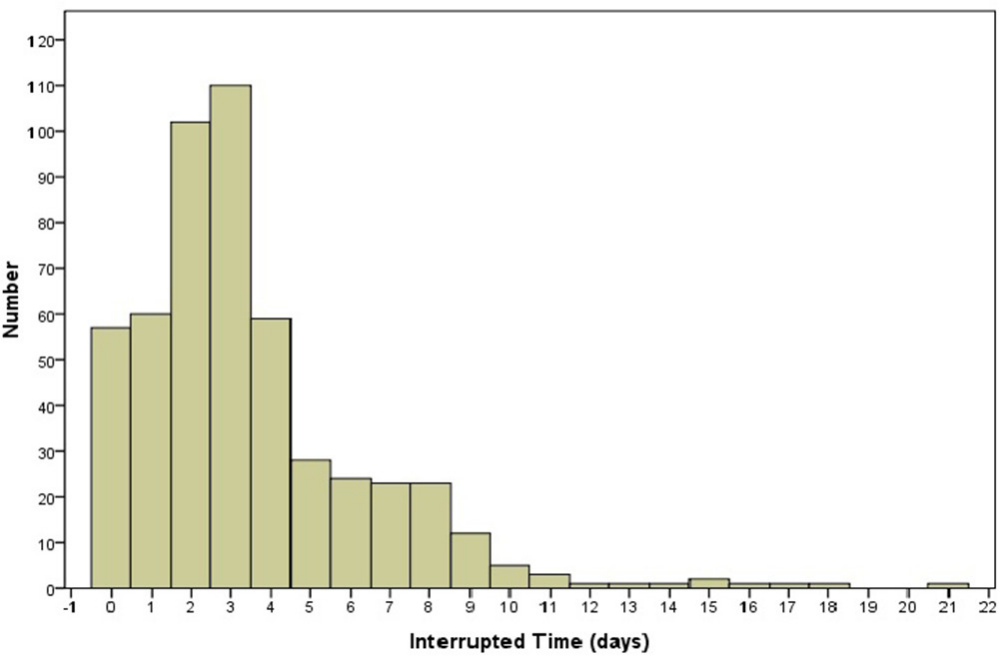
Histogram of interrupted time during intensity modulated radiotherapy in NPC patients

The distributions of the IT values are shown in Figure 1. To analyze the impact of IT, P25 (2 days), P50 (3 days), and P75 (4 days) values are used in this study. According to the corresponding cut-off values, all patients in the study were divided into two different groups. For example, to dichotomize the time parameter at IT-P25 (2 days), two groups were created for comparison (IT >2 days *vs*. IT ≤2 days).

### The influence of survival outcome

#### The interrupted time of 2 days

**Figure 2:**
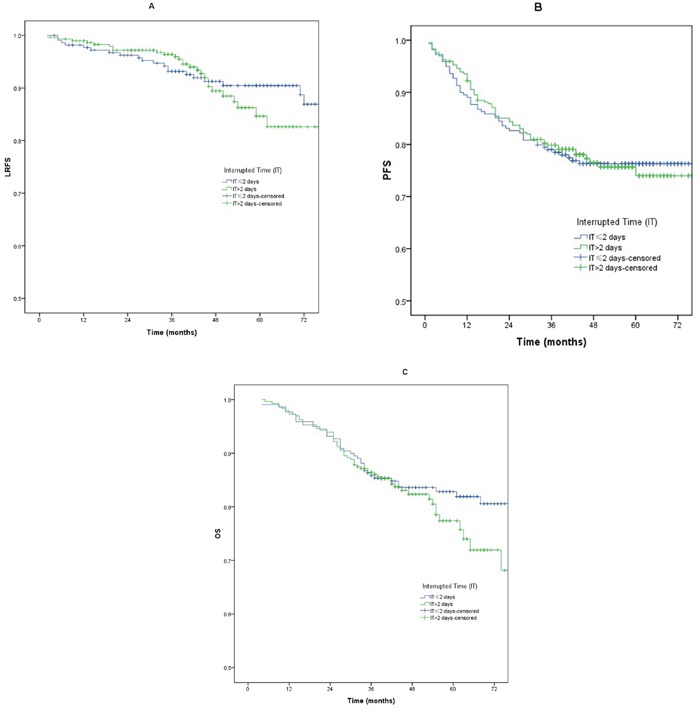
**A-C**. Loco-regional failure-free survival (LRFS), progression free survival (PFS) and overall survival (OS) between patients with a IT >2 days and those with a IT ≤ 2 days.

There were no significant differences for PFS, LRFS, and OS between patients with a IT >2 days and those with a IT ≤2 days (*p* >0.05) (Table 2). The LRFS, PFS, and OS curves between patients with a IT >2 days and those with a IT ≤2 days are shown in Figure 2A–2C.

**Table 2:** The rates of locoregional recurrence-free survival (LRFS), progress-free survival (PFS), and overall survival (OS) among different cut-off values of interrupted time

Group	LRFS		p value	PFS		p value	OS		p value
	1-Year	3-Year		1-Year	3-Year		1-Year	3-Year	
IT(>2 days vs ≤ 2 days)	99.0% vs 97.7%	96.4% vs 94.2%	0.662	93.6% vs 89.0%	79.9% vs 79.0%	0.972	98.0% vs 97.7%	86.4% vs 85.8%	0.308
IT(>3 days vs ≤ 3 days)	99.5% vs 98.2%	95.9% vs 94.9%	0.817	90.9% vs 90.9%	76.2% vs 81.4%	0.078	98.4% vs 97.6%	84.9% vs 87.5%	0.040
IT(>4 days vs ≤ 4 days)	99.2% vs 98.2%	95.5% vs 94.9%	0.765	92.9% vs 91.2%	72.1% vs 81.9%	0.010	97.6% vs 97.4%	80.8% vs 87.9%	0.002

Abbreviations: LRFS= locoregional recurrence-free survival, PFS=progress-free survival, OS= overall survival, CI= confidence interval, IT= interrupted time.

#### The interrupted time of 3 days

**Figure 3:**
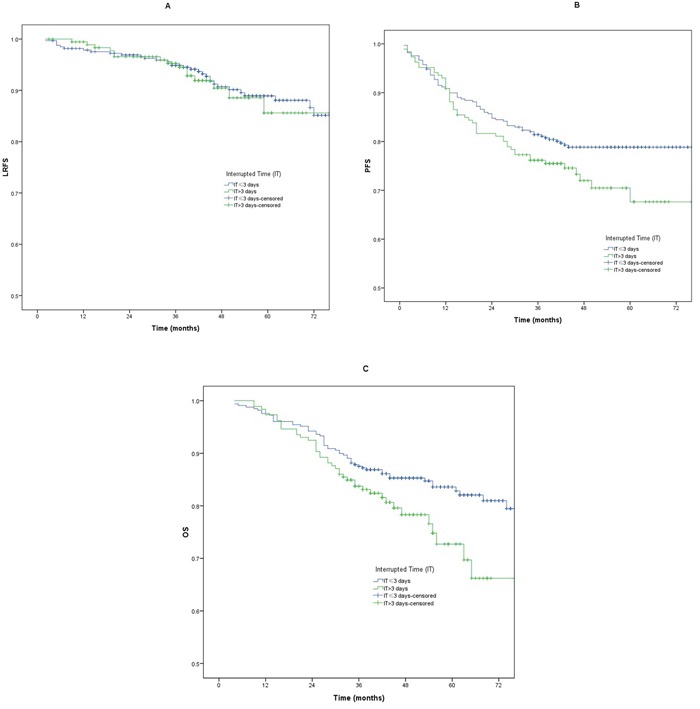
**A-C**. Loco-regional failure-free survival (LRFS), progression free survival (PFS) and overall survival (OS) between patients with a IT >3 days and those with a IT ≤3 days.

There were no significant differences for PFS and LRFS between patients with a IT > 3 days and those with a IT ≤3 days (*p* >0.05) (Table 2). The 3-year OS analysis revealed significant difference between patients with a IT >3 days and those with a IT ≤3 days (72.7% *vs* 83.65%, *p* =0.04). The LRFS, PFS, and OS curves between patients with a IT >3 days and those with a IT ≤3 days are shown in Figure 3A–3C.

#### The interrupted time of 4 days

**Figure 4:**
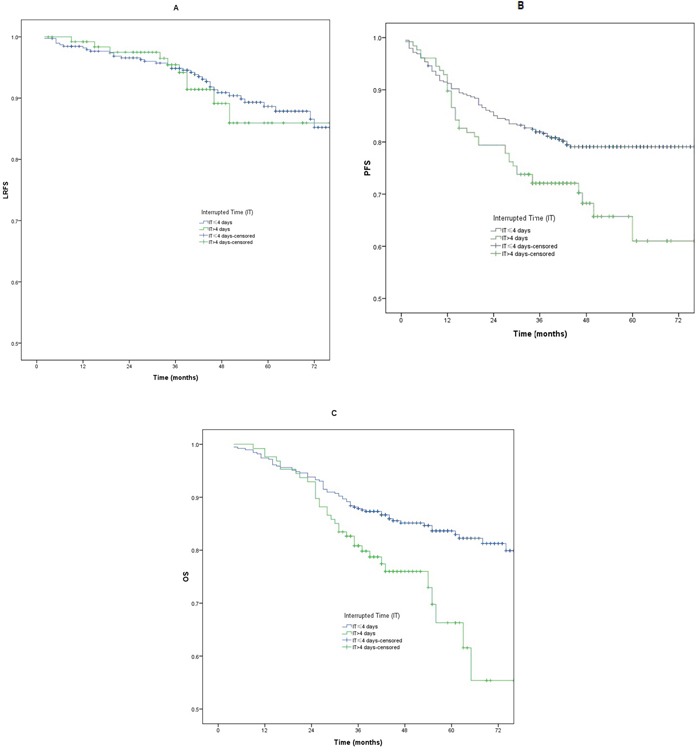
**A-C**. Loco-regional failure-free survival (LRFS), progression free survival (PFS) and overall survival (OS) between patients with a IT >4 days and those with a IT ≤4 days.

There was no significant difference for LRFS between patients with a IT >4 days and those with a IT ≤4 days (*p* >0.05) (Table 2). The 3-year PFS and OS analysis revealed significant difference between patients with a IT >4 days and those with a IT ≤4 days (72.1% *vs* 81.9%, *p* =0.01; 80.8% *vs* 87.9%, *p* =0.002). The LRFS, PFS, and OS curves between patients with a IT >4 days and those with a IT ≤4 days are shown in Figure 4A–4C.

### Prognostic factors

To determine the independent prognostic factors, we conducted multivariate analysis using variables in this study, including age (≥44 years *vs* <44 years), WHO type, T classification, N classification, concurrent chemotherapy (yes *vs* no) and IT (>4 days *vs* ≤4days). Multivariate analysis revealed that age (≥44 years *vs* <44 years), T classification, N classification, and IT (> 4 days *vs* ≤4days) acted as significant predictive factors for OS (*p* =0.001, *p* =0.000, *p* =0.005, and *p* =0.011, respectively), and PFS (*p* =0.014, *p* =0.000, *p* =0.000, and *p* =0.043, respectively). At the same time, it should be noted that N classification was the only prognostic factor for LRFS (*p* =0.015) (Table 3).

**Table 3:** Multivariate analysis of locoregional recurrence-free survival (LRFS), progress-free survival (PFS), and overall survival (OS)

Factor	LRFS hazard ratio	P-value	PFS hazard ratio	P-value	OS hazard ratio	P-value
Year(<44 y *vs* ≥44y)	0.809 (0.455-1.411)	0.473	1.584 (1.099-2.285)	0.014	2.026(1.343-3.057)	0.001
Gender	0.585 (0.280-1.221)	0.153	0.709 (0.466-1.129)	0.148	0.795 (0.481-1.315)	0.372
concurrent chemotherapy (yes *vs* no)	1.033 (0.431-2.478)	0.941	0.644 (0.364-1.140)	0.131	0.661 (0.368-1.187)	0.166
T classification	1.234 (0.910-1.673)	0.175	1.492 (1.214-1.832)	0.000	1.604 (1.274-2.018)	0.000
N classification	1.578 (1.093-2.278)	0.015	1.683 (1.282-2.210)	0.000	1.515 (1.133-2.027)	0.005
IT (>4 d *vs* ≤4d)	1.047 (0.512-2.142)	0.900	1.488 (1.012-2.188)	0.043	1.741 (1.135-2.668)	0.011

Abbreviations: LRFS= locoregional recurrence-free survival, PFS=progress-free survival, OS= overall survival, CI= confidence interval, IT= interrupted time.

## DISCUSSION

Several previous studies about conventional radiotherapy showed that the prolonged time of radiotherapy may result in poorer rates of PFS [5–9]. Although the IMRT technology has been extensively utilized in the treatment of NPC, the influence of the interruption during IMRT on survival outcomes of NPC patients was rarely studied. Li PJ and his colleagues [10] retrospectively analyzed 321 NPC patients receiving IMRT and found that various cutoff values of IT (>1 day *vs* ≤1 day, >3 days *vs* ≤3 days, and, >6 days *vs* ≤6 days) had no significant association with PFS. Within the range of the overall radiotherapy time observed in our study, the cutoff values of IT (>2 days *vs* ≤2 days, and, >3 days *vs* ≤3 days) didn't have significant association with PFS. But IT (>4 days *vs* ≤4 days) was the independent prognostic factors for PFS in NPC patients receiving IMRT. The result differences of these two studies may be caused by different sample size and distribution of IT.

Now the clinical data about the effect of the duration of radiotherapy interruptions on loco-regional control for NPC patients mainly derived from conventional radiotherapy [5–9]. And the prolonged time of radiotherapy and treatment interruptions may result in poorer loco-regional control in NPC patients receiving conventional radiotherapy [5–9]. Compared with conventional radiotherapy, our study and two previous studies [10, 11] illustrated that the duration of radiotherapy interruptions had no significant association with loco-regional control in NPC patients receiving IMRT. The following differences between conventional radiotherapy and IMRT may explain the disparity. Firstly, the planed overall time of conventional radiotherapy is at least 7 weeks (≥35 fractions). While the prescribed course of the intensity-modulated radiotherapy with simultaneous integrated boost (IMRT-SIB) can be completed in about 6 weeks (30 to 33 fractions). The application of IMRT-SIB may reduce the number of proliferated tumor cells by shortening the total treatment time without decreasing total dose. Secondly, the fractionated dose of IMRT for PTVnx (2.26–2.27Gy/fraction) is larger than that of conventional radiotherapy (1.8–2Gy/fraction), which may contribute to suppress reparation of sub-lethal damage. Thirdly, using the commonly accepted assumption of an α/β ratio equal to 10, the calculated EQD_2_ of IMRT for PGTVnx (69.96–74.09Gy) were 71.5Gy–75.8Gy, which is larger than the conventional dose of conventional radiotherapy (70Gy). The improved total dose of IMRT may contribute to compensate the effect of the duration of radiotherapy interruptions for loco-regional control.

According to several prospective randomized trials [12–14], concurrent chemoradiotherapy is superior to radiotherapy alone for the management of stage II–IVB NPC. Although the benefit of neoadjuvant chemotherapy or adjuvant chemotherapy is still open to debate, neoadjuvant chemotherapy or adjuvant chemotherapy is commonly prescribed for most patients with III-IV stage disease at our hospital. But it is worth noting that concurrent chemotherapy had no significant impact on PFS, LRFS or DFFS in our study and the previous study [10]. This may be caused by the fact that the subgroup receiving concurrent chemotherapy contained a significantly higher proportion of patients with locally advanced NPC. Moreover, the main technology of radiotherapy used in these prospective randomized trials was conventional radiotherapy, while all patients from our study received IMRT-SIB. The role of concurrent chemoradiotherapy in patients receiving IMRT-SIB is still controversial.

The majority of patients with “prolongation” can complete radiotherapy within several days after the scheduled date. About 25% of patients had prolonged treatment to more than 4 days. Our study has identified a cut-off point of IT (> 4 days vs ≤4days), which may affect PFS and OS in NPC patients receiving IMRT. Tumor clonogen repopulation starts around the third to fifth week after the beginning of radiotherapy [15]. The stimulation of radiotherapy may decrease the tumor clonogen doubling time from about 60 days to 4 days by the middle of treatment [15]. Further studies with longer follow-up and larger populations are urgently needed to confirm the time-point and duration time of radiotherapy interruption, which may affect survival outcomes in clinical practice.

In conclusion, the IT of more than 4 days may have significant associations with survival outcomes in patients with nasopharyngeal cancer receiving IMRT. We should minimize the interruption of radiotherapy caused by any reason as much as possible.

## MATERIALS AND METHODS

### Patients

Review of the database of Cancer Hospital of Guangxi Medical University identified 520 newly diagnosed patients with NPC receiving definitive IMRT between January 2007 and December 2011. The study was approved by the institutional review board. The pretreatment workup included nasopharyngeal fiberoptic endoscopy, and contrast-enhanced magnetic resonance imaging of the head and neck, chest X-ray/computed tomography (CT), abdominal ultrasound/CT, and bone scan (79 patients didn't receive bone scan due to machinery faults, the lack of radioactive drug, and patients with early-stage disease.). All patients were restaged using the 7th edition of American Joint Committee on Cancer (AJCC). The pathological type was confirmed according to the 2005 World Health Organization (WHO) classification of tumors. Patients with a history of previous malignant tumors (5 patients) were excluded.

### Treatment

#### Radiotherapy

After thermoplastic mask fitting and CT-based simulation, all of the patients received high-dose IMRT. The gross tumor volume of nasopharynx (GTVnx) and the involved cervical lymph nodes (GTVnd) were determined based on magnetic resonance imaging (MRI), clinical, and endoscopic findings. The enlarged retropharyngeal nodes together with primary gross tumor volume were outlined as the GTVnx on the IMRT plans. High-risk subclinical tumor volume (CTV-1) was defined as the area from 0.5-1.0 cm outside the GTV and a site that involves potential sites of local infiltration. The low-risk subclinical tumor volume (CTV2) was defined as the margin from 0.5-1.0 cm around CTV1 and the lymph node draining area (Levels II, III, IV and V). The planning tumor volume for nasopharynx (PTVnx), the involved cervical lymph nodes (PTVnd), high-risk subclinical area (PTV1), and low-risk subclinical area (PTV2) were defined by adding a 3 mm margin to the GTVnx, GTVnd, CTV1, and CTV2. In IMRT planning, we used a simultaneously integrated boost IMRT technique. The moderate hypofractionation scheduled IMRT delivered 69.96-74.09Gy at 30-32 fractions to PTVnx, 66.00-72.32Gy at 30-32 fractions to PTVnd, 60-62Gy at 30-31 fractions to PTV1, and 54.0-55.8Gy at 30-31 fractions to PTV2.

#### Chemotherapy

In this study, 457 patients received platinum-based chemotherapy, including neoadjuvant chemotherapy, concurrent chemotherapy and adjuvant chemotherapy. Neoadjuvant chemotherapy and concurrent chemotherapy were given every 3 weeks. And adjuvant chemotherapy was administered every 4 weeks. The common regimens of neoadjuvant chemotherapy included TPF (docetaxel, cisplatin, and 5-fluorouracil) and PF (cisplatin and 5-fluorouracil). The regimens of concurrent chemotherapy consisted of PF (cisplatin and 5-fluorouracil) and single cisplatin. The regimen of adjuvant chemotherapy was PF (cisplatin and 5-fluorouracil).

### Assessment and follow-up

Patients were evaluated weekly during radiotherapy. After treatment completion, follow-ups occurred every 3 months for the first 2 years, every 6 months from the second through the fifth year and annually thereafter. Each follow-up included physical examination, nasopharyngeal fiberoptic endoscopy, enhanced MRI of the head and neck, chest X-ray/CT, abdominal ultrasound/CT, and bone scan. Additional tests were ordered whenever there was any indication from results of examinations.

### Statistical analysis

Chi-square test was used for comparing the differences of clinical characteristics between patients with a IT >4 days and those with a IT ≤4 days. The primary endpoints of this study were loco-regional failure-free survival (LRFS), progression free survival (PFS) and overall survival (OS). Time to events in this study was determined from the end of treatment to the event of interest or the end of follow-up. Patients were censored if no events occurred by last follow-up. IT was defined as radiotherapy treatment time minus time scheduled for the patients to complete the prescribed course of radiotherapy. IT was analyzed as the dichotomous variable in univariate analysis and multivariate analysis, using the lower quartile (P25), the median (P50) and upper quartile (P75) values as cut-off points [10]. The rates of LRFS, PFS, and OS were calculated with the Kaplan-Meier method, and the differences among groups were compared with a log-rank test. The Cox regression proportional hazard model using the backward elimination method was used for multivariate analyses and evaluating the prognostic factors for LRFS, PFS, and OS. Two-tailed values of *p* <0.05 were considered significant. Statistical Package for Social Sciences (SPSS version 20.0) software was used for statistical analyses.

### Ethical statement

This retrospective study was approved by the Ethics Committee of Cancer Hospital of Guangxi Medical University. Patient informations were all anonymous.

## References

[1] Wei WI, Sham JS (2005). Nasopharyngeal carcinoma. Lancet.

[2] Chang ET, Adami HO (2006). The enigmatic epidemiology of nasopharyngeal carcinoma. Cancer Epidemiol Biomarkers Prev.

[3] Blanchard P, Lee A, Marguet S, Leclercq J, Ng WT, Ma J, Chan AT, Huang PY, Benhamou E, Zhu G, Chua DT, Chen Y, Mai HQ (2015). Chemotherapy and radiotherapy in nasopharyngeal carcinoma: an update of the MAC-NPC meta-analysis. Lancet Oncol.

[4] Sun X, Su S, Chen C, Han F, Zhao C, Xiao W, Deng X, Huang S, Lin C, Lu T (2014). Long-term outcomes of intensity-modulated radiotherapy for 868 patients with nasopharyngeal carcinoma: an analysis of survival and treatment toxicities. Radiother Oncol.

[5] Kwong DL, Sham JS, Chua DT, Choy DT, Au GK, Wu PM (1997). The effect of interruptions and prolonged treatment time in radiotherapy for nasopharyngeal carcinoma. International journal of radiation oncology, biology, physics.

[6] He XY, Dai CH, Huan SL (2002). Effect of the treatment time and the Timing of interruption on local control of T1-T2 NPC in radiotherapy. Fudan Univ. J Med Sci.

[7] Du XH, Tang ZZ, Zheng X, Zhu Y, Wang YZ (2003). Study on the influence to the therapeutic effects with prolonged period of radiotherapy in nasopharyngeal carcinoma. Cancer Research and Clinic.

[8] Wu SX, Zhao C, Lu TX, Chen M, Xie FY, Cui NJ (2000). Influence of prolonged overall treatment time and interruptions on outcome in continuous radiotherapy for nasopharyngeal carcinoma. Chin J Cancer.

[9] Tan BX, Zhang YW, Hu CS, WU YR, Feng Y (1998). The time dose response of nasopharyngeal carcinoma treated with conventional radiotherapy. Chin J Radiat Oncol.

[10] Li PJ, Jin T, Luo DH, Shen T, Mai DM, Hu WH, Mo HY (2015). Effect of prolonged radiotherapy treatment time on survival outcomes after intensity-modulated radiation therapy in nasopharyngeal carcinoma. PLoS One.

[11] Su SF, Han F, Zhao C, Chen CY, Xiao WW, Li JX, Lu TX (2011). The effect of overall treatment time on local control in nasopharyngeal carcinoma patients treated with intensity modulated radiation therapy. Natl Med J China.

[12] Chen QY, Wen YF, Guo L, Liu H, Huang PY, Mo HY, Li NW, Xiang YQ, Luo DH, Qiu F, Sun R, Deng MQ, Chen MY (2011). Concurrent chemoradiotherapy vs radiotherapy alone in stage II nasopharyngeal carcinoma: phase III randomized trial. J Natl Cancer Inst.

[13] Lin JC, Jan JS, Hsu CY, Liang WM, Jiang RS, Wang WY (2003). Phase III study of concurrent chemoradiotherapy versus radiotherapy alone for advanced nasopharyngeal carcinoma: positive effect on over-all and progression-free survival. J Clin Oncol.

[14] Chan AT, Teo PM, Ngan RK, Leung TW, Lau WH, Zee B, Leung SF, Cheung FY, Yeo W, Yiu HH, Yu KH, Chiu KW, Chan DT (2002). Concurrent chemotherapy-radiotherapy compared with radiotherapy alone in locoregionally advanced nasopharyngeal carcinoma: progression-free survival analysis of a phase III randomized trial. J Clin Oncol.

[15] Withers HR, Taylor JM, Maciejewski B (1988). The hazard of accelerated tumor clonogen repopulation during radiotherapy. Acta Oncol.

